# Effect of eHealth Interventions on Body Image of Patients With Cancer: Systematic Review

**DOI:** 10.2196/55564

**Published:** 2025-01-09

**Authors:** Guohong Huang, Rongrong Wu, Xiuzhi Xu, Yongxia Song, Rong Zheng, Xi Chen, Jingfang Hong

**Affiliations:** 1 School of Nursing Anhui Medical University Hefei China; 2 Nursing Department The Second People's Hospital of Fuyang City Fuyang China

**Keywords:** body image, cancer, eHealth, systematic review, quality of life, physical symptom, emotional distress, review, mobile phone

## Abstract

**Background:**

Body image issues are prevalent among individuals diagnosed with cancer, leading to detrimental effects on their physical and psychological recovery. eHealth has emerged as a promising approach for enhancing the body image of patients with cancer.

**Objective:**

The purpose of this study was to evaluate the effectiveness of eHealth interventions on body image and other health outcomes (quality of life, physical symptoms, and emotional distress) among patients with cancer. In addition, the acceptability, engagement, and challenges of eHealth interventions were also assessed.

**Methods:**

A total of 11 databases were searched, encompassing PubMed; Embase; Web of Science; MEDLINE (via Ovid); Scopus; the Cochrane Library; CINAHL (via EBSCO); OpenGrey; and 3 prominent Chinese repositories: China National Knowledge Infrastructure, China Wanfang Database, and China VIP Database. The search dates were from the inception of the database to September 25, 2024. The inclusion criteria for this study encompassed research that used randomized controlled trials (RCTs) and quasi-experiments (QEs) to examine the effectiveness of eHealth interventions for patients with cancer. The methodological quality of RCTs and QEs was evaluated using the Cochrane risk of bias tool and the Joanna Briggs Institute Critical Evaluation Checklist, respectively. The review adhered to the PRISMA (Preferred Reporting Items for Systematic Reviews and Meta-Analyses) guidelines and was registered with PROSPERO.

**Results:**

There were 3548 studies reviewed, and 7 studies were selected. Three studies were RCTs and 4 were QEs, involving a total of 512 patients. Evidence of efficacy for eHealth interventions targeting body image and other health outcomes (physical symptoms and emotional distress) was mixed. Nevertheless, our findings indicate that there was no notable enhancement in quality of life resulting from eHealth interventions. A total of 5/7 (71%) studies reported the acceptability of eHealth interventions among patients with cancer, and patients perceived eHealth interventions as acceptable. However, the difficulty of operating the software, determination of the most effective course of treatment, and time constraints emerged as the primary challenges associated with electronic interventions.

**Conclusions:**

The implementation of eHealth interventions has the potential to enhance body image, physical symptoms, and emotional distress in patients with cancer. Researchers should undertake more rigorous experiments in the future to elucidate the effectiveness of eHealth and address pertinent concerns.

## Introduction

Cancer is an escalating global public health issue. Worldwide, the number of cancer diagnoses is swiftly increasing, projected to rise from 19.3 million in 2020 to approximately 21.6 million by 2030 [1,2]. Fortunately, with the innovations of technologies in early diagnosis and cancer treatment, it is more likely that patients with cancer will live longer and have a better prognosis [3]. However, cancer and subsequent treatments may lead to a range of physical changes including, but not limited to, cosmetic changes (eg, hair loss, scarring, and swelling), sensory changes (eg, pain and numbness), and dysfunction (eg, dysphagia, dysarthria, and impotence), which can severely affect the patients of body image [4].

It has been widely acknowledged that an individual’s body image is a complex construct encompassing perceptions, thoughts, feelings, and behaviors related to the entire body [4]. Body image disturbance in adult patients with cancer manifests as self-perceived dissatisfaction with physical alterations, functional impairments, and psychological distress resulting from changes in appearance or functionality [5]. According to the American Cancer Society, it is estimated that between 31% and 67% of the approximately 3.1 million survivors of breast cancer in the United States have experienced distress related to body image issues [6]. The prevalence of body image disturbance in patients with head and neck cancer has been reported to be as high as 74% [7]. A study has demonstrated that body image concerns are not limited to patients with breast, head, and neck cancer. These concerns significantly affect a substantial proportion of patients with cancer and persist throughout their long-term survival [4]. These findings are concerning, as prospective research has indicated that poor body image can result in elevated levels of anxiety, depression, and sexual and intimacy concerns, as well as an increased risk of mortality [8,9].

Therefore, there is a growing concern about the importance of body image in the diagnosis and treatment of patients with cancer. According to a report published by Breast Cancer Care, a prominent UK charity, there was an urgent need for increased support for women undergoing breast cancer treatment to address their body image concerns [10]. The international skincare and make-up workshop, “Look Good, Feel Better,” offers instructional sessions on makeup techniques to address eyebrow loss and eyelash loss in women [11]. However, traditional face-to-face intervention may struggle to achieve the ideal intervention effect due to time constraints, geographical barriers, lack of medical resources, and high costs. The emergence of eHealth may overcome the shortcomings of these traditional interventions.

eHealth is an accessible health information delivery strategy that provides information and health services through the internet and related technologies [12-14]. eHealth interventions are recognized for their numerous advantages. First, the privacy and confidentiality of patients are ensured during the implementation [15]; second, multiple patients can attend simultaneously and repeatedly, and the fidelity of the intervention is not affected by time constraints and different interveners [15]; and finally, eHealth interventions can be considered as a less time-consuming and cost-effective method of delivering interventions.

Given the diverse advantages of eHealth interventions, it has been gradually applied to address body image issues among patients with cancer, but the findings were still controversial. Sherman et al [16] revealed that web-based psychological intervention, a structured writing exercise, significantly improved body image distress among patients with breast cancer. However, Høyer et al [17] found that teleconferencing did not improve the body image of patients with breast cancer undergoing radiotherapy. A recent investigation indicated that the group using a smartphone app for exercise did not demonstrate a significant improvement in body image among patients with cancer when compared to the traditional exercise group [18]. Therefore, the purpose of this study was to evaluate the effectiveness of eHealth interventions for improving body image among patients with cancer, considering the methodological quality of the most recent clinical trials on the topic, and other secondary outcomes (quality of life, physical symptoms, and emotional distress) were also investigated. Finally, the acceptability, engagement, and challenges of relevant eHealth interventions were discussed.

## Methods

### Overview

This systematic review has been registered on the PROSPERO platform (CRD42023388898). It was performed in accordance with the PRISMA (Preferred Reporting Items for Systematic Reviews and Meta-Analyses) guidelines [19] (protocol for conducting systematic reviews) and following the population, intervention, comparison, outcomes, and study (PICOS) design [20].

### Search Strategy

A total of 11 databases were searched, encompassing PubMed; Embase; Web of Science; MEDLINE (via Ovid); Scopus; the Cochrane Library; CINAHL (via EBSCO); OpenGrey; and three prominent Chinese repositories: China National Knowledge Infrastructure, China Wanfang Database, and China VIP Database. The search dates were from the inception of the database to September 25, 2024. The reference lists of pertinent systematic reviews were scrutinized to identify any additional studies that could be incorporated [11,21-26]. The search form included the truncations and synonyms for the following terms: neoplasms, cancer, onco*, internet-delivered, app, eHealth, mHealth, smartphone, telephone, text message*, body image, self-image (Multimedia Appendix 1 provides a comprehensive search strategy for each database).

### Inclusion and Exclusion Criteria

The studies that met the following criteria were included in this review: (1) the study population comprised adults aged 18 years and older, who had been diagnosed with cancer and were either undergoing or had completed cancer treatment; (2) the intervention in the studies were related to eHealth interventions, which encompassed various modes of communication such as SMS text messaging, phone, email, app, web, smartphone app, and videoconferencing; (3) the comparison groups were standard care or control intervention (access to the internet without specific guidance from intervention personnel); (4) the primary outcome of the study was body image, which was assessed using standardized, scientifically validated, and reliable psychometric instruments; (5) the studies design included randomized controlled trial (RCT) or quasi-experimental (QE) studies; and (6) the language of the study was Chinese or English.

Studies were excluded for the following reasons: (1) the experimental group involved a combination of eHealth and face-to-face interventions; (2) body image was used as a secondary outcome; (3) the study included study protocols without outcomes, review, nonclinical study, meta-analysis, and so on; (4) the full text cannot be obtained; and (5) studies were repeated publications.

### Study Selection

The research team deliberated and reached a consensus regarding the search terms. Literature was retrieved by 2 researchers (GH and RW) and subsequently imported separately into the document management software NoteExpress (Beijing Aiqin Haile Technology Co, Ltd). Title and abstract screening and full-text screening were performed independently by 2 investigators (GH and RW) in accordance with the same inclusion and exclusion criteria. Any disagreements were initially addressed and resolved by 2 researchers. Otherwise, a third researcher (XX) was involved to ensure agreement was reached. Finally, GH reviewed all studies to determine inclusion or exclusion.

### Data Collection Process

Excel data extraction tables were developed according to PRISMA guidelines [19]. The information mainly included (1) general research information: author, year, country, participants, design type, study duration, mean age, and sample size; (2) intervention and control group details: intervention platform, method, intervention, and follow-up time; and (3) outcomes: evaluation tool and primary and secondary outcomes.

### Quality Assessment

The methodological quality of each included RCT was evaluated using the Cochrane Risk of Bias (RoB) Tool [27]. Quasi-randomized studies were assessed for RoB using the Joanna Briggs Institute (JBI) Critical Appraisal Checklist [28]. RoB was independently assessed by authors RW (3 studies) and XX (3 studies). The JBI tool was independently evaluated by authors RZ (3 studies) and XC (3 studies). Discrepancies were addressed through collaborative discussion by the review team. Finally, GH checked all assessments to ensure accuracy.

### Data Synthesis

The heterogeneity in methods and outcomes precluded the use of meta-analysis. Instead, a narrative overview of the findings from the included studies was presented alongside a tabular summary of the extracted data. Study outcomes were divided into primary outcomes (body image) and other health outcomes (quality of life, physical symptoms, and emotional distress).

## Results

### Study Characteristics

**Figure 1:**
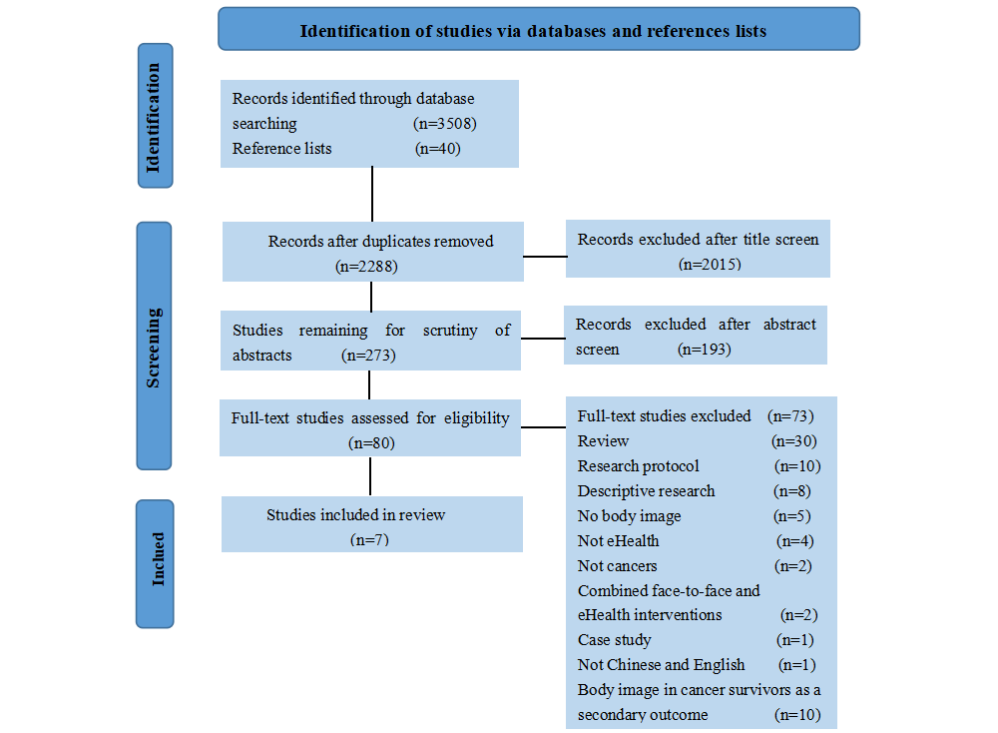
PRISMA flowchart describing study identification and selection process. PRISMA: Preferred Reporting Items for Systematic Reviews and Meta-Analyses.

After eliminating duplicates and conducting a screening process, 7 full-text studies were identified (Figure 1), including a total of 512 participants. The general characteristics of each study are reported in Multimedia Appendix 2 [16,29-34]. In terms of research methodology, 3 (43%) out of the 7 studies were RCTs and the rest (n=4, 57%) were QE studies.

Based on the study population, 4 out of 7 studies were conducted on women diagnosed with breast cancer, accounting for 57% of the total number of studies [16,29-31]. As for study location, the included studies were conducted in Europe (n=1, 14%) [32], the United States (n=2, 29%) [31,33], Australia (n=2, 29%) [16,34], or Asia (n=2, 29%) [29,30].

### Overview of eHealth Interventions

A total of 3 (43%) studies were conducted through a website or web-based app [16,30,34]. All others were conducted by SMS text messages, telephone, or video intervention [29,31-33]. The intervention methods included psychological intervention (n=5, 71%) [16,30,32-34], health education intervention (n=1, 14%) [29], and physical exercise intervention (n=1, 14%) [31]. The duration of intervention ranged from 30 minutes to 3 months. The practitioners involved in the intervention involved doctors [16], oncologists [29,34], peer mentors [31], psychologists [29,30,32,33], and health education specialists [29].

Due to the extensive inclusion criteria and resultant heterogeneity of outcomes, we reported outcomes involving 4 subject-related categories under eHealth interventions: body image [16,29-34], quality of life [32], physical symptoms (fatigue [29,31] and sexual function [31]), and emotional distress (anxiety and depression [16,30-32], self-efficacy [30], and self-compassion [16]).

### Primary Outcome

All of the study outcomes measured patients’ body image [16,29-34]. Nine body image measures, validated in patients with cancer, were used: Body Image Concern Inventory [29], Body Image Scale [16,30-32], Body Mindfulness Questionnaire [32], Body Image States Scale [34], Body Image Coping Skills Inventor [33], IMAGE-HN (Inventory to Measure and Assess image disturbance—Head and Neck) [33], Body Appreciation Scale [16], the Body Appreciation Scale-2 Short Form [34], and Multidimensional Body-Self Relations Questionnaire [34]. The intervention methods included psychological intervention via the internet, app, videoconference, telephone- or text-based health education intervention [16,29,30,32-34], physical exercise intervention using activity monitors, and videoconference [31]. A total of 6 (86%) studies reported a significant effect of the intervention [16,29-31,33,34]. A 3-month, internet-based, peer-moderated physical activity (PA) intervention (Pink Body Spirit) for young patients with breast cancer showed that participants reported an improvement in body image at 3 months after the intervention [31]. A 1-week, web-based brief writing intervention for patients with cancer demonstrated that participants reported a significant improvement in body image 1 week after the intervention [34]. A 7-week app health education intervention was conducted for patients with breast cancer. The results showed that the body image of the intervention group was significantly improved after 7 weeks of intervention [29]. A 6-week, internet-based, mindfulness-based stress reduction intervention for patients with breast cancer showed significant improvement in body image at 6 weeks after the intervention [30]. A 3-month, internet-based structured writing intervention for patients with breast cancer showed a significant reduction in body image concerns within the intervention group after 3 months [16]. A brief cognitive behavioral intervention based on a video telemedicine platform was conducted for patients with head and neck cancer for 3 months. The results showed that body image was significantly improved after 3 months of intervention [33]. Of them, 4 studies [16,31,33,34] tracked the effects of the intervention at multiple time points (≥2 time points), but not all of these studies achieved the intended.

### Other Health Outcomes

#### Quality of Life

Quality of life was assessed as a secondary outcome in 14% (n=1) of studies. This study used a quality-of-life scale that has been validated in patients with cancer: the European Organization for Research and Treatment of Cancer Quality of Life Questionnaire Core 30, with higher scores reflecting better quality of life. This study was based on smartphone-based group physical and psychological therapy, and the treatment effect on quality of life at 5 weeks after the intervention was statistically insignificant [32].

#### Physical Symptoms

In total, 2 (29%) studies investigated physical symptoms, including fatigue and sexual function. Two studies showed significant reductions in fatigue, including one video remote intervention [31] and one eHealth education intervention [29]. A study focusing on female sexual function indicated that a PA intervention using remote video peer support did not yield improvements in sexual function among young patients with breast cancer [31].

#### Emotional Distress

A total of 4 (57%) studies investigated the outcome of emotional distress, including anxiety and depression, self-compassion, and self-efficacy [16,30-32]. The results from 4 separate studies evaluated the prevalence of anxiety and depression. Notably, 2 studies indicated that a remote, video-based, peer-led intervention focused on exercise detection and an internet-based, mindfulness-based stress reduction intervention were effective in reducing anxiety symptoms; however, they did not produce significant enhancements in depressive symptoms [30,31]. In contrast, the remaining study reported that the intervention was successful in improving both anxiety and depression levels for participants with breast cancer and lymphedema [16]. Yet another study indicated that smartphone-based physical and psychological interventions did not improve anxiety and depression [32]. A study reported significant improvements in self-compassion with a structured web-based writing exercise intervention [16]. The Internet-Mindfulness-Based Stress Reduction intervention greatly improved self-efficacy [30].

#### Acceptability

A total of 5 (71%) studies reported the acceptability of eHealth interventions among patients with cancer [16,30-32,34]. Participants rated videoconferencing as acceptable and promising [31]. Participants reported that internet-based teaching methods allowed them to feel more relaxed [30]. A total of 88% of respondents felt that internet-based interventions were worthwhile [16]. Alternatively, participants perceived the intervention via the internet as beneficial [34]. A study indicated that 92.5% of participants expressed a higher level of satisfaction with psychosomatic therapy delivered via smartphone [32].

#### Engagement

A total of 3 (43%) studies evaluated participants’ involvement with the intervention through the completion of modules and use tracking [16,31,32]. A study revealed that 88% of participants in the intervention group completed all 6 components of the web writing intervention [16]. Another study showed that 92.5% of participants participated in at least 4 video sessions of 15 physical smartphone-based interventions [32]. Weiner et al [31] found that more than 85% of people wore an exercise monitoring device on at least 75% of intervention days.

#### Challenges

A total of 3 (43%) studies reported challenges faced during the intervention process [16,30,31]. A psychosocial intervention program delivered via the internet has indicated that certain older patients with cancer encountered challenges in using the software, such as difficulties in accessing courses and experiencing audio issues during course playback [30]. An RCT reported that the web-based My Changed Body psychosocial intervention was delivered only once, and therefore, it could not be concluded that the optimal number of administrations of the writing for maximum benefit [16]. At the same time, participants in another study said that videoconferencing may take more time to coordinate than email and SMS text messaging because of the potential time conflicts involved in dealing with daily events [31].

#### RoB Assessment

**Figure 2:**
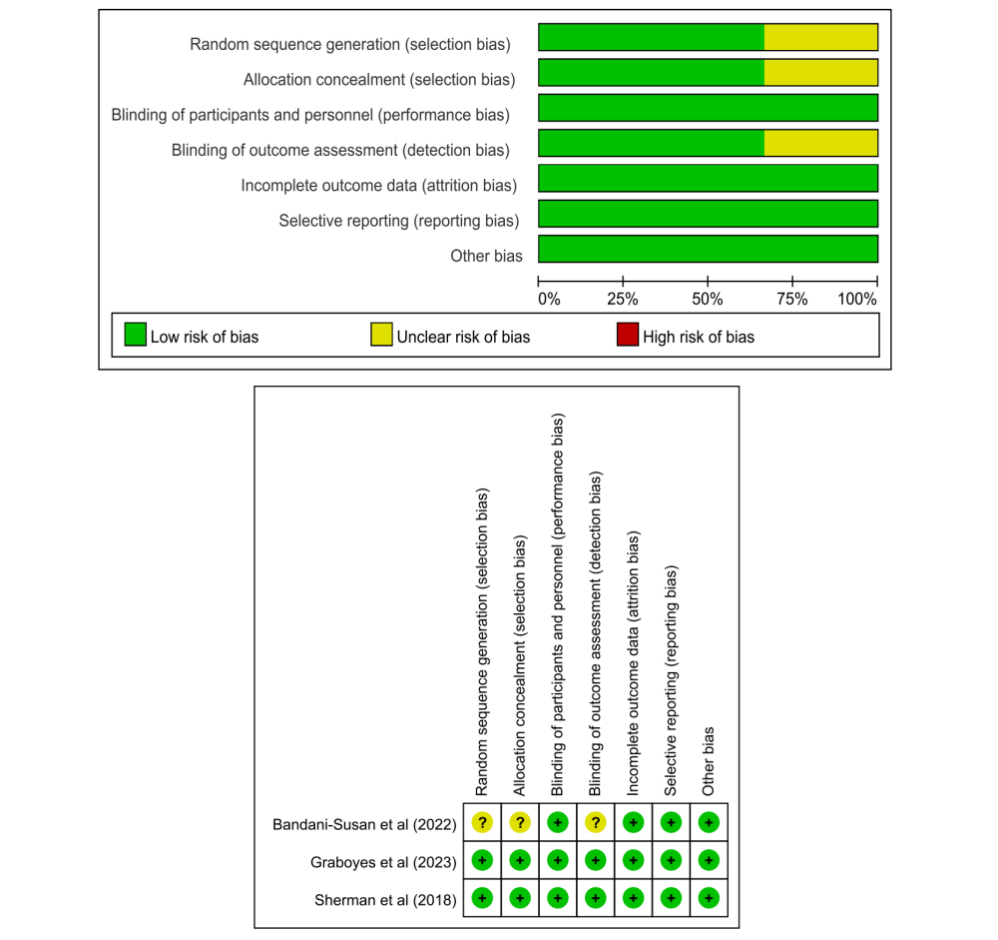
Cochrane risk of bias scores (% low, unclear, and high risk) across bias domains.

This study used the Cochrane Collaboration’s RoB assessment tool, as well as the JBI tool for quality assessment of RCTs and quasi-randomized studies, respectively. The final results are shown in Figure 2 [16,29,33] and Table 1. Two studies within the RCT were assessed and found to exhibit a low RoB across all 6 evaluated dimensions [16,33]. Selection bias was evaluated as unclear in a study due to insufficient information [29]. Moreover, one study had some detection bias due to a lack of blinding [29]. In addition, regarding quasi-randomized studies, the comparability of baseline data was not clearly articulated for most studies (n=3, 75%) [30-32]. As for the two groups of interventions, only one study explicitly stated that all other measures received by each group were the same [30]. Regarding multivariate measures of outcome indicators, most of the studies (n=3, 75%) implemented diversified measures of outcome indicators before and after the intervention [31,32,34]. With respect to the treatment of follow-up data, all studies reported missing data; however, only one study failed to address this issue with statistical methods [30]. For further details, please refer to the Multimedia Appendices 3 and 4. In addition, the study was performed in accordance with the PRISMA guidelines (Multimedia Appendix 5).

**Table 1:** Joanna Briggs Institute Critical Appraisal Checklist for quasi-experimental evaluation.

Author (year)	①^a^	②^b^	③^c^	④^d^	⑤^e^	⑥^f^	⑦^g^	⑧^h^	⑨^i^
Weiner et al (2023) [31]	Y^j^	NC^k^	NA^l^	Y	Y	Y	Y	Y	Y
Chang et al (2022) [30]	Y	N^m^	Y	Y	N	N	Y	Y	Y
Brkic et al (2024) [34]	Y	Y	N	Y	Y	Y	Y	Y	Y
Grossert et al (2023) [32]	Y	NC	NA	Y	Y	Y	Y	Y	Y

^a^Is it clear in the study what is the “cause” and what is the “effect” (ie, there is no confusion about which variable comes first)?

^b^Were the participants included in any similar comparisons?

^c^Were the participants included in any comparisons receiving similar treatment/care other than the exposure or intervention of interest?

^d^Was there a control group?

^e^Were there multiple measurements of the outcome both before and after intervention/exposure?

^f^Was follow-up complete and if not, were differences between groups in terms of their follow-up adequately described and analyzed?

^g^Were the outcomes of participants included in any comparisons measured in the same way?

^h^Were outcomes measured in a reliable way?

^i^Was appropriate statistical analysis used?

^j^Y: yes.

^k^NC: not clear.

^l^NA: not applicable.

^m^N: no.

## Discussion

### Principal Findings

In light of the growing prevalence of eHealth interventions, their application in patients with cancer has been on the rise. To our knowledge, this study was the first systematic review to evaluate the effects of eHealth interventions on body image in individuals diagnosed with cancer. This study aimed to scrutinize published research on eHealth interventions for body image in patients with cancer and critically evaluate the effectiveness of these interventions. In addition, other secondary outcomes (quality of life, physical symptoms, and emotional distress) also were explored. Finally, the acceptance, engagement, and challenges faced by patients with cancer during eHealth intervention were also assessed.

From the perspective of research characteristics, the majority(4/7, 57%) of studies involved female patients with breast cancer, possibly attributed to the higher incidence rate of breast cancer in women or their generally heightened concern toward appearance and body image compared to men [35]. Recently, body image interventions in patients with prostate cancer and head and neck cancer have also emerged [36,37], indicating that body image problems in patients with other cancer types are also receiving scholars’ attention. We observed that the majority(5/7, 71%) of the studies incorporated in this review were conducted in high-income countries, probably because high-income countries have a higher penetration of smartphones and computers and more stable networks, which was more conducive to the development of research. According to 2023 International Telecommunication Union statistics, there has been a noticeable increase in mobile network use in low- and middle-income countries [38]. Therefore, it is not difficult to carry out research based on eHealth interventions in limited-income countries in the future.

The intervention outcomes, encompassing body image, physical symptoms, and emotional distress, yielded mixed results. The eHealth-based interventions in this study included psychological intervention [16,30,32-34], health education intervention [29], and physical exercise intervention [31]. Our analysis revealed that a majority (6/7, 86%) of the eHealth intervention studies demonstrated substantial effects on body image. In previous studies, face-to-face or group psychotherapy, psychoeducation, and PA have shown positive effects on the perception of body imagery among patients with cancer, but limitations in time, medical resources, and geographic location make it difficult to implement widespread interventions [11,39]. However, these problems were well addressed using electronic information interventions [40]. Although this study found that eHealth-based interventions can have a positive effect, the effect of the interventions waned as the duration of follow up increased. Given the availability of resources, it is recommended that future research explore the most serious time points of body image distress in patients with cancer and that targeted interventions may achieve the best results at the lowest possible economic cost. In addition, we demonstrated that a group-based physical and psychological intervention significantly enhances the appreciation of body awareness among survivors of cancer [32]. Therefore, we suspect that resources permitting, future studies may achieve better results if multiple intervention modalities are implemented simultaneously. Given that only a single study within the reviewed literature assessed the quality of life, the findings indicated that the enhancement in quality of life resulting from eHealth interventions did not reach statistical significance [32]. The limited follow-up period may be a contributing factor to the lack of significant differences observed in the study. Given that the duration of an intervention is a crucial determinant of its effectiveness, it is essential to carefully consider this aspect during the design phase of the program. A large, cross-country RCT study demonstrated the significant benefits of remote monitoring in mitigating symptom management and emotional distress in patients with cancer [41]. However, in this study, the outcomes of interventions targeting physical symptoms and emotional distress had mixed results. These studies did not specify which component was most influential in alleviating symptoms. The effective intervention of emotional distress was mainly based on the positive perspective to provide psychological support for patients [30,31].

From the viewpoint of attitudes toward eHealth interventions, a majority( 37/40, 92.5%) of patients with cancer expressed satisfaction, acceptance, and perceived value and welcomed these interventions. However, only 3 studies reported patient participation in intervention completion [16,31,32], and none of the included studies examined the economic costs of the intervention. This suggests that future research should delve into strategies for sustaining the appeal of intervention programs while ensuring patient participation, taking into account economic costs. In addition, the study explored the challenges of eHealth interventions, including the difficulty of operating the software, determination of the most effective course of treatment, and time conflicts. Some implications for future research can be obtained. First, it is suggested that multidisciplinary cooperation is needed in eHealth intervention to evaluate the physical and mental conditions of patients with cancer and formulate reasonable and scientific intervention programs. Second, eHealth interventions may pose a challenge for patients with cancer in areas with poorly developed network communications. Therefore, robust technical support and patient education provided by professional staff are essential. Finally, considering the time constraints that patients with cancer often encounter, eHealth interventions can offer greater flexibility, enabling patients to engage in them during their leisure time.

### Study Limitations

The study findings were meticulously reviewed and reported in strict adherence to the PRISMA guidelines. However, the study does have certain limitations. First, although we conducted a comprehensive search as much as possible, some studies may have been missed due to language limitations. Second, the heterogeneity in intervention methods, frequency, timing, and assessment tools among the included studies precludes the application of a meta-analysis program for synthesizing their results. Consequently, narrative data synthesis was deemed to be the most suitable approach. Finally, among the 7 studies included in this study, 4 studies were QEs, and the sample sizes of 2 studies were relatively modest, which may affect the research conclusion.

### Conclusions

In conclusion, this research indicates that eHealth interventions may be beneficial for improving body image, physical symptoms, and emotional distress in individuals with cancer. To further validate their effectiveness, future high-quality RCTs are warranted. In the future, more multidisciplinary teams are needed to explore the availability, acceptability, effectiveness, and cost of different eHealth interventions to improve the health outcomes of patients with cancer.

## Appendix

Multimedia Appendix 1 Search strategy for each database.

Multimedia Appendix 2 Summary of a systematic review of electronic health (eHealth) interventions on body image in patients with cancer.

Multimedia Appendix 3 Cochrane risk-of-bias supporting evidence for randomized controlled trials.

Multimedia Appendix 4 Joanna Briggs Institute risk-of-bias supporting evidence for quasi-experiments.

Multimedia Appendix 5 PRISMA (Preferred Reporting Items for Systematic Reviews and Meta-Analyses) checklist for effect of electronic health interventions for the body image of patients with cancer.

## Abbreviations

JBI: Joanna Briggs Institute
PA: physical activity
PICOS: population, intervention, comparison, outcomes, and study
PRISMA: Preferred Reporting Items for Systematic Reviews and Meta-Analyses
QE: quasi-experiment
RCT: randomized controlled trial
RoB: risk of bias
