# Supplementary material for: Effect of eHealth Interventions on Body Image of Patients With Cancer: Systematic Review
Source: J Med Internet Res. 2025 Jan 9;27:e55564. doi: 10.2196/55564 (PMC11757978; doi:10.2196/55564)
Supplement: Multimedia Appendix 1 [file jmir_v27i1e55564_app1.docx]

| **Database** | **Search strategy** | **Search results** |
| --- | --- | --- |
| Pubmed  (1986 to September 2024) | #1:telemedicine[MeSH Terms] OR (Internet-delivered OR eHealth OR mHealth OR telecommunication OR telehealth OR mobile OR smartphone OR wireless OR handheld OR cell Phone? OR app? OR application? OR software? OR electronic* OR phone? OR telephone? OR mobile technolog* OR web-based OR Web page OR Internet OR social media OR text messag* OR email*)[Title/Abstract]  #2:neoplasms [MeSH Terms] OR (neoplasm* OR cancer* OR malignan* OR neoplas* OR tumor* OR tumour* OR onco* OR adenocarcinoma* OR carcinoma* OR tumeur* OR new growth OR phyma* OR ambury* OR anbury* OR metastat* OR glioma* OR lymphoma* OR myeloma* OR leukemia* OR leucaemia*) [Title/Abstract]  #3:body Image[MeSH Terms] OR (self-image OR body dissatisfaction OR self-concept OR body experience OR body schema*)  [Title/Abstract]  #4:#1 AND #2 AND #3 | **151** |
| Web of science  （2006 to September 2024） | #1:Title:(telemedicine OR Internet-delivered OR eHealth OR mHealth OR telecommunication OR telehealth OR mobile OR smartphone OR wireless OR handheld OR cell Phone? OR app? OR application? OR software? OR electronic* OR phone? OR telephone? OR mobile technolog* OR web-based OR Web page OR Internet OR social media OR text messag* OR email*)  #2:Title:（neoplasms OR neoplasm* OR cancer* OR malignan* OR neoplas* OR tumor* OR tumour* OR onco* OR adenocarcinoma* OR carcinoma* OR tumeur* OR new growth OR phyma* OR ambury* OR anbury* OR metastat* OR glioma* OR lymphoma* OR myeloma* OR leukemia* OR leucaemia*）  #3:Title:(body Image OR self-image OR body dissatisfaction OR self-concept OR body experience OR body schema*)  #4:#1 AND #2 AND #3 | **14** |
| Medline(via Ovid)  （1975 to September 2024） | S1:(MM "Telemedicine+")  S2:AB(Internet-delivered OR eHealth OR mHealth OR telecommunication OR telehealth OR mobile OR smartphone OR wireless OR handheld OR cell Phone? OR app? OR application? OR software? OR electronic* OR phone? OR telephone? OR mobile technolog* OR web-based OR Web page OR Internet OR social media OR text messag* OR email*）  S3:S1 OR S2  S4:(MM“Neoplasms+”)  S5:AB(neoplasm* OR cancer* OR malignan* OR neoplas* OR tumor* OR tumour* OR onco* OR adenocarcinoma* OR carcinoma* OR tumeur* OR new growth OR phyma* OR ambury* OR anbury* OR metastat* OR glioma* OR lymphoma* OR myeloma* OR leukemia* OR leucaemia*)  S6:S4 OR S5  S7:(MM "Body Dissatisfaction") OR (MM "Body Image+") OR (MM "Self Concept+"）  S8: AB (self-image or body experience or body schema* )  S9:S7 OR S8  S4:S3 AND S6 AND S9 | **728** |
| Scopus  (1986 to September 2024) | (ABS ("body image" OR self-image OR "body dissatisfaction" OR self-concept OR "body experience" OR "bodyschema*" ) ) AND ( ABS ( neoplasms OR neoplasm* OR cancer* OR malignan*  OR neoplas* OR tumor* OR tumour* OR onco* OR adenocarcinoma* OR carcinoma* OR tumeur* OR "new growth" OR phyma* OR ambury* OR anbury* OR metastat* OR glioma* OR lymphoma* OR myeloma* OR adenocarcinoma* OR leukemia* OR leucaemia* ) ) AND ( ABS ( telemedicine OR internet delivered OR ehealth OR mhealth OR telecommunication OR telehealth OR mobile OR smartphone OR wireless OR handheld OR "cell phone?" OR app? OR application? OR software? OR electronic* OR phone? OR telephone? OR "mobile technolog*" OR web-based OR "web page" OR internet OR "social media" OR "text messag*" OR email* ) ) | **164** |
| Embase  (1986 to September 2024) | #1:telemedicine[MeSH] OR (Internet-delivered OR eHealth OR mHealth OR telecommunication OR telehealth OR mobile OR smartphone OR wireless OR handheld OR cell Phone? OR app? OR application? OR software? OR electronic* OR phone? OR telephone? OR mobile technolog* OR web-based OR Web page OR Internet OR social media OR text messag* OR email*)[Title/Abstract]  #2:neoplasms [MeSH Terms] OR (neoplasm* OR cancer* OR malignan* OR neoplas* OR tumor* OR tumour* OR onco* OR adenocarcinoma* OR carcinoma* OR tumeur* OR new growth OR phyma* OR ambury* OR anbury* OR metastat* OR glioma* OR lymphoma* OR myeloma* OR adenocarcinoma* OR leukemia* OR leucaemia*) [Title/Abstract]  #3:(body Image[MeSH] OR  body dissatisfaction[MeSH] OR self-concept[MeSH] )OR (self-image OR body experience OR body schema*)  [Title/Abstract]  #4:#1 AND #2 AND #3 | **2050** |
| CINAHL (via EBSCO)  (Inception to September 2024) | S1:TX telemedicine OR TI Digital Health OR TI Internet-delivered OR TI Telephone OR TI telecommunication OR TI smartphone OR TI Electronic Mail OR TI Distance Counseling OR TI smartphone OR TI wireless OR TI mobile technolog OR TI web-based  S2:TX neoplasms OR TI neoplasm OR TI cancer* OR TI malignan* OR TI neoplas OR TI tumor OR TI tumour OR TI onco OR TI adenocarcinoma OR TI carcinoma OR TI new growth OR TI phyma  S3:TX body Image OR TI Social Comparison OR TI Body Dissatisfaction OR TI Diagnostic Self Evaluation OR TI Egocentrism OR TI Self-Assessment OR TI Self Disclosure OR TI Self Efficacy OR TI Self-Compassion OR TI Sense of Coherence  # S1 AND S2 AND S3 | **109** |
| The Cochrane Library  (Inception to September 2024) | #1:(telemedicine) OR (mHealth):ti,ab,kw OR (Mobile Health):ti,ab,kw OR (eHealth):ti,ab,kw OR (Telehealth):ti,ab,kw OR (Tele-Intensive Care):ti,ab,kw OR (Tele Care):ti,ab,kw OR (Tele-Referrals):ti,ab,kw OR (Virtual Medicine):ti,ab,kw  #2:(neoplasms) OR (Benign Neoplasms):ti,ab,kw OR (Neoplasias):ti,ab,kw OR (Tumors):ti,ab,kw OR (Cancer):ti,ab,kw OR (adenocarcinoma*):ti,ab,kw OR ( carcinoma**):ti,ab,kw OR ( carcinoma*):ti,ab,kw OR ( new growth):ti,ab,kw OR (adenocarcinoma*):ti,ab,kw OR (phyma*):ti,ab,kw OR ( ambury* ):ti,ab,kw OR ( anbury*):ti,ab,kw OR ( carcinoma*):ti,ab,kw  #3:(body Image) OR (Body Schemas):ti,ab,kw OR (Body Identity):ti,ab,kw OR (Body Images):ti,ab,kw OR (body dissatisfaction):ti,ab,kw OR (self-concept):ti,ab,kw OR (self-image):ti,ab,kw OR (body experience):ti,ab,kw OR (body schema):ti,ab,kw  #4:#1 AND #2 AND #3 | **161** |
| China National Knowledge Infrastructure  (1979 to September 2024) | AB(telemedicine OR Internet-delivered OR eHealth OR mHealth OR telecommunication OR telehealth OR mobile OR smartphone OR wireless OR handheld OR cell Phone? OR app? OR application? OR software? OR electronic* OR phone? OR telephone? OR mobile technolog* OR web-based OR Web page OR Internet OR social media OR text messag* OR email*)AND AB(theme（neoplasms OR neoplasm* OR cancer* OR malignan* OR neoplas* OR tumor* OR tumour* OR onco* OR adenocarcinoma* OR carcinoma* OR tumeur* OR new growth OR phyma* OR ambury* OR anbury* OR metastat* OR glioma* OR lymphoma* OR myeloma* OR leukemia* OR leucaemia*）  AND AB(theme(body Image OR self-image OR body dissatisfaction OR self-concept OR body experience OR body schema*) | **113** |
| China Wanfang Database  (1989 to September 2024) | ( neoplasm* OR cancer* OR malignan* OR neoplas* OR tumor* OR tumour* OR onco* OR adenocarcinoma* OR carcinoma* OR tumeur* OR new AND growth OR phyma* OR ambury* OR anbury* OR metastat* OR glioma* OR lymphoma* OR myeloma* OR adenocarcinoma* OR leukemia* OR leucaemia* )[Title or Keyword] AND ( self-image OR body  dissatisfaction OR self-concept OR body  experience OR body  image  )[Title or Keyword] AND ( therapy OR treatment OR intervention OR counseling OR psychotherapy )[Title or Keyword] | **1** |
| VIP Journal Integration Platform  (1977 to September 2024) | R ( neoplasm* OR cancer* OR malignan* OR neoplas* OR tumor* OR tumour* OR onco* OR adenocarcinoma* OR carcinoma* OR tumeur* OR new AND growth OR phyma* OR ambury* OR anbury* OR metastat* OR glioma*  OR lymphoma* OR myeloma* OR adenocarcinoma* OR leukemia* OR leucaemia* )AND R ( self-image OR body  dissatisfaction OR self-concept OR body  experience OR body  image  )AND R( therapy OR treatment OR intervention OR counseling OR psychotherapy ) | **8** |
| OpenGrey  (Inception to September 2024) | neoplasm* OR cancer* OR malignan* OR tumour* AND body Image OR self-image OR body dissatisfaction OR self-concept OR body experience OR body schema* | **9** |

Note: MM: MeSH; AB: abstract; M: title or keyword; ABS:abstract;R:abstract
