# Supplementary material for: Effect of eHealth Interventions on Body Image of Patients With Cancer: Systematic Review
Source: J Med Internet Res. 2025 Jan 9;27:e55564. doi: 10.2196/55564 (PMC11757978; doi:10.2196/55564)
Supplement: Multimedia Appendix 2 [file jmir_v27i1e55564_app2.docx]

Table S1**.** Summary of a systematic review of electronic health(eHealth) interventions on body image in patients with cancer(1)

| **Author**  **/year**  **/Country** | **Participants** | **Design/sample size (n)/ retention rate (%)** | **Experimental group** | | **Control group**  **Contents** | **Outcomes measured:**  **Instrument of measurement** | **Results** |
| --- | --- | --- | --- | --- | --- | --- | --- |
|  |  |  | **Intervention duration/**  **time for measures** | **Contents** |  |  |  |
| Bandani‑Susan[1]/  2022/ Iran | women with breast cancer | RCT/Total:46IG (n=23-19),CG(n=23-19)/82.6% | 7 weeks/baseline,7 week | *Educational messages* were sent, including week 1, introduction and generalities about the disease and complications of treatment; week  3, cancer-related fatigue; and weeks 5–7 appearance and body image. | 1 month after completing the post-test,educational text messages were sent for 2 weeks. | Fatigue: Cancer Fatigue Scale  Body Image: Body Image Concern Inventory | Cancer fatigue and body image were significantly improved in the intervention group after intervention, while there was no statistical significance in the control group. After intervention, the average score of body image was significantly different between the two groups. |
| Sherman  [2]/2018/ Australia | women with breast cancer | RCT/Total:304  IG(n=155-142),CG(n=149-137)/91.8% | 30-minute/baseline and 1 week, 1 month, and 3 months | *A Web-based structured writing exercise writing activity***.** First, participants were instructed to write freely a distressing event related to their body after breast cancer. Next, they continued writing about their body image after cancer treatment guided by five self-compassionate prompts, concluding with a narrow focus on the personal situation (total of six writing parts) | A single 30-minute Web-based writing activity without any self-compassionate  focused writing prompts. | Body Image: Body Image Scale; Body Appreciation Scale  Self-compassion: Self-Compassion Scale–Short Form  Depression and anxiety: Depression, Anxiety, and Stress Scales  Appearance investment: Appearance Schemas Inventory | Compared with the control group, the intervention group significantly improved body image and significantly increased body appreciation and self-compassion scores. Treatment effects were maintained at 1 month (BID and body appreciation) and 3 months (body appreciation) after the intervention. Psychological distress was significantly reduced (depression for 1 month; 1 week and 1 month of anxiety). |

| **Author**  **/year**  **/Country** | **Participants** | **Design/sample size (n)/ retention rate (%)** | **Experimental group** | | **Control group**  **Contents** | **Outcomes measured:**  **Instrument of measurement** | **Results** |
| --- | --- | --- | --- | --- | --- | --- | --- |
|  |  |  | **Intervention duration/**  **time for measures** | **Contents** |  |  |  |
| Weiner[3]/  2023  /USA | younger breast cancer survivors (18-54 years old) | Quasi-experimental study(one-group pretest-posttest design)/Total:34-31/91.8% | 3-month/baseline, 3-months ,6 months | Participants were mailed a Fitbit Charge 3（an accelerometer-based *activity monitor*）and completed a brief *Zoom video* call with research staff to set-up their Fitbit.Participants completed 6 video chat or phone sessions with their peer mentor over 3-month and interacted with their peer mentor and other participants through a private Fitbit Community at least weekly. Between scheduled sessions, peer mentors used real-time Fitbit data to identify participants in need of additional support to increase their exercise. | N/A | Body image: Body Image Scale  Sexual function: Female Sexual Function Index  Fatigue, Anxiety, Depression, Emotional support: PROMIS Cancer v1.0; PROMIS v2.0 measure  Physical activity: ActiGraph GT3X+ (ActiGraph, LLC) | From baseline to 3-months, participants increased time spent in objectively measured MVPA(moderate-to-vigorous physical activity), strength, and flexibility exercises, and reported meaningful improvements to body image, fatigue, anxiety, and emotional support. |
| Chang[4] /2022/ Taiwan, China | women with breast cancer | Quasi-experimental study/Total:72 IG(n=40-41(from waitlist control group),CG(n=32-26)/93.1% | 6-week /baseline and post intervention within a week | The frequency and duration of mindfulness training were six 2-h weekly group coaching sessions using *internet-delivered learning with Microsoft Teams.*. The informal training was conducted through home practice using mindfulness-guided *cloud-based applications such as YouTube*. | They had manuals for managing symptoms and oral instructions on health and hygiene education. | Body Image: Body Image Scale  Depression, Anxiety, Stress: Depression, Anxiety, and Stress Scale  Self-efficacy: General Self-Efficacy Scale | Internet-Mindfulness-Based Stress Reduction  (iMBSR) did not significantly improve depression and stress between groups, iMBSR could improve anxiety with medium effect sizes. Significant benefits were found for body image and self-efficacy, with large effect sizes. |

| **Author**  **/year**  **/Country** | **Participants** | **Design/sample size (n)/ retention rate (%)** | **Experimental group** | | | **Control group**  **Contents** | **Outcomes measured:**  **Instrument of measurement** | **Results** |
| --- | --- | --- | --- | --- | --- | --- | --- | --- |
|  |  |  | **Intervention duration/**  **time for measures** | | **Contents** |  |  |  |
| Graboyes [5]/2023/ USA | Adult head and neck survivors with body image-related distress | RCT/Total:44 IG(n=24), CG(n=20)/100% | 3-month/baseline, 1-week, 1-month, and 3-months post-intervention | The BRIGHT(Building a Renewed  Image after Head & neck cancer Treatment)was a consisting of 5 weekly 60-minute sessions delivered one-on-one by a licensed clinical psychologist via a video telemedicine platform. | | we designed match BRIGHT’s dose (five weekly sessions)and delivery method (video-based telemedicine) while  not providing the behavior change mechanism in BRIGHT | Body image: Body Image Coping Skills Inventory, IMAGE-HN [Inventory to Measure and Assess image disturbance–Head and Neck] | We provided preliminary evidence that BRIGHT effectively reduces maladaptive body image coping relative to control group and demonstrate that the reduction of maladaptive body image coping (particularly avoidance and appearance fixing) decreases body image among HNC survivors. |
| Grossert [6]/2023/ Switzerland | women with cancer | Quasi-experimental study(one-group pretest-posttest design)/Total:42-39/92.86% | 5 weeks/baseline, 6 weeks followed by a pre-intervention assessment, and the post-intervention assessment | Patients participated in six group BPT sessions, 90 min each. In parallel, they received daily homework *via* smartphone. The manualized group BPT was carried out in small groups (range of 5–7 patients) and was provided by trained psychotherapists. | | N/A | Body image:Body Image Scale, Body Mindfulness Questionnaire,  Anxiety, Depression: Hospital Anxiety and Depression Scale, Multidimensional Mood Questionnaire,  Quality of life: European Organization for Research and Treatment of Cancer , Short Form Health Survey | Although the body image of patients in the intervention group was not statistically significant, a strong intervention effects and significant improvements were observed with regard to the appreciation of body awareness. |

| **Author**  **/year**  **/Country** | **Participants** | **Design/sample size (n)/ retention rate (%)** | **Experimental group** | | | **Control group**  **Contents** | **Outcomes measured** | **Results** |
| --- | --- | --- | --- | --- | --- | --- | --- | --- |
|  |  |  | **Intervention duration/**  **time for measures** | | **Contents** |  |  |  |
| Brkic[7]/  2024/ Australia | women with cancer | Quasi-experimental study/Total:201  IG(n=100-8), CG(n=101-6)/ 7.0% | 1-week/baseline, Immediately after the intervention, post intervention within a week | This study tested the feasibility and efficacy of two iterations of a low-intensity Web-based writing intervention.  Study 1: Intervention included three 15-min sessions that occurred throughout a week-long  period (Days 1, 4, and 7).Participants were introduced to the concept of body functionality appreciation, and were guided through six categories of possible body functions, with each session covering two functions.  Study 2: Participants completed a modified 15-minute single-session version.  . | | Study 1: The control group included three 15-min creative writing sessions on  Days 1, 4 and 7.  Study2: Participants were asked to write for a mini  mum of 15-min, and provide specific details about their day, including  what tasks they completed. | Study 1: Trait Body Appreciation: The Body Appreciation Scale-2 Short Form, Trait Body Dissatisfaction: Multidimensional Body-Self Relations Questionnaire,  Trait distress: Depression, Anxiety and Stress Scale.  Study 2: Body appreciation: Body Appreciation Scale-2  Functionality Appreciation: Functionality Appreciation Scale  Body dissatisfaction: Body Image States Scale | Study 1: In the context of a cancer population, three sessions of writing may be too burdensome.As such, Study 2 trialled a single-session writing intervention.  Study 2: Participants in intervention group had  larger associated effect sizes for improvements in state body functionality appreciation and distress, compared to control group. |

Abbreviations:RCT:Randomized Controlled Trial;IG:Intervention Group;CG:Control Group;QOL:Quality of Life;HRQoL, health-related quality of life;N/A, not applicable

Table S2. Summary of a systematic review of electronic health(eHealth) interventions on body image in patients with cancer(2)

| Author /year | Age,years | Treatment approach | Mode of delivery | Implementor | Body  image | Quality of life | Sexual function | Anxiety | Self-compassion |
| --- | --- | --- | --- | --- | --- | --- | --- | --- | --- |
|  |  |  |  |  |  |  | Fatigue | Depression | Self-efficacy |
|  |  |  |  |  | *P-value* | *P-value* | *P-value* | *P-value* | *P-value* |
| Weiner 2023 [3] | 43.1 (5.5) | Peer-led intervention | Zoom Video Calling | Peer mentor | <.001 | - | 0.889 | < 0.034 | - |
|  |  |  |  |  |  |  | < 0.001 | < 0.121 | - |
| Chang 2022 [4] | 49.61 (12.03) | Mindfulness-based stress reduction | Applications | Psychologists | 0.003 | - | - | 0.041 | - |
|  |  |  |  |  |  |  | - | 0.918 | 0.004 |
| Bandani‑Susan 2022 [1] | 46.34 (9.96) | Health education intervention | WhatsApp Messenger | Health education specialists, psychologists and oncologists. | 0.002 | - | - | - | - |
|  |  |  |  |  |  |  | 0.005 | - | - |
| Grossert 2023 [6] | - | Body psychotherapy | Smartphone-based interventions | Psychologists | 0.339 | 1 | - | 0.554 | - |
|  |  |  |  |  |  |  | - | 0.673 | - |
| Sherman 2018 [2] | 57.50 (8.98) | Structured writing exercises | Web | Clinicians | 0.002 | - | - | 0.007 | ＜0.001 |
|  |  |  |  |  |  |  | - | 0.001 | - |
| Graboyes 2023 [5] | 63 [41-80] | Cognitive behavioral therapy | Video teleconferencing | Psychologists | 0.029 | - | - | - | - |
|  |  |  |  |  |  |  | - | - | - |
| Brkic 2024 [7] | 57.12 (12.71) | Brief online writing intervention | Web | Oncologists | <.001 | - | - | - | - |
|  |  |  |  |  |  |  | - | - | - |

**References**

1. Bandani-Susan B, Montazeri A, Haghighizadeh MH, Araban M. The effect of mobile health educational intervention on body image and fatigue in breast cancer survivors: a randomized controlled trial. Ir J Med Sci 2022 Aug;191(4):1599-1605. [doi: [10.1007/s11845-021-02738-5](https://link.springer.com/article/10.1007/s11845-021-02738-5)] [Medline:[34370166](https://pubmed.ncbi.nlm.nih.gov/34370166/)]
2. Sherman KA, Przezdziecki A, Alcorso J, Kilby CJ, Elder E, Boyages J, et al. Reducing body image-related distress in women with breast cancer using a structured online writing exercise: results from the my changed body randomized controlled trial. J Clin Oncol. 2018;36(19):1930

-40. [[FREE Full text](https://www.sci-hub.ru/10.1200/JCO.2017.76.3318)][doi:[10.1200/JCO.2017.76.3318](https://www.sci-hub.ru/10.1200/JCO.2017.76.3318)][Medline:[29688834](https://pubmed.ncbi.nlm.nih.gov/29688834/)]

1. Weiner LS, Nagel S, Irene Su H, Hurst S, Levy SS, Arredondo EM, et al. A remotely delivered, peer-led intervention to improve physical activity and quality of life in younger breast cancer survivors. J Behav Med 2023 Aug;46(4):578-593. [[FREE Full text](https://www.ncbi.nlm.nih.gov/pmc/articles/PMC9735111/)][doi: [10.1007/s10865-022-00381-8](https://pubmed.ncbi.nlm.nih.gov/36479658/)][Medline:[36479658](https://pubmed.ncbi.nlm.nih.gov/36479658/)]
2. Chang YC, Chiu CF, Wang CK, Wu CT, Liu LC, Wu YC. Short-term effect of internet-delivered mindfulness-based stress reduction on mental health, self-efficacy, and body image among women with breast cancer during the COVID-19 pandemic. Front Psychol 2022 Oct ;13:949446. [[FREE Full text](https://www.ncbi.nlm.nih.gov/pmc/articles/PMC9640939/)][doi: [10.3389/fpsyg.2022.949446](https://www.frontiersin.org/journals/psychology/articles/10.3389/fpsyg.2022.949446/full)][Medline:[36389600](https://pubmed.ncbi.nlm.nih.gov/36389600/)]
3. Graboyes EM, Kistner-Griffin E, Hill EG, Maurer S, Balliet W, Williams AM, et al. Mechanism underlying a brief cognitive behavioral treatment for head and neck cancer survivors with body image distress. Support Care Cancer. 2023;32(1):32.[doi: [10.1007/s00520-023-08248-7](https://link.springer.com/article/10.1007/s00520-023-08248-7)][Medline:[38102496](https://pubmed.ncbi.nlm.nih.gov/38102496/)]
4. Grossert A, Meffert C, Hess V, Rochlitz C, Pless M, Hunziker S, et al. Group-based body psychotherapy improves appreciation of body awareness in post-treatment cancer patients: a non-randomized clinical trial. Front Psychol. 2023;14:956493. [[Free Full text](https://www.ncbi.nlm.nih.gov/pmc/articles/PMC10117640/)][doi :[10.3389/fpsyg.2023.956493](https://www.frontiersin.org/journals/psychology/articles/10.3389/fpsyg.2023.956493/full)][Medline:[36479658](https://pubmed.ncbi.nlm.nih.gov/37089722/)]
5. Brkic E, Prichard I, Daly A, Dudley S, Beatty L. Testing the efficacy of a brief online writing intervention on body image and distress in female cancer survivors. Patient Educ Couns. 2024;127:108356. [doi: [10.1016/j.pec.2024.108356](https://www.sciencedirect.com/science/article/pii/S0738399124002234?via%3Dihub)][Medline:[38944983](https://pubmed.ncbi.nlm.nih.gov/38944983/)]
