# Supplementary material for: Effect of eHealth Interventions on Body Image of Patients With Cancer: Systematic Review
Source: J Med Internet Res. 2025 Jan 9;27:e55564. doi: 10.2196/55564 (PMC11757978; doi:10.2196/55564)
Supplement: Multimedia Appendix 3 [file jmir_v27i1e55564_app3.docx]

| Author/  Year | Random sequence  generation | | Allocation  concealment | | Blinding of  participants  and personnel | | Blinding  of outcomes | | Incomplete outcome data | | Selective reporting | | Other bias | |
| --- | --- | --- | --- | --- | --- | --- | --- | --- | --- | --- | --- | --- | --- | --- |
|  | Judgment | Supporting  evidence | Judgment | Supporting  evidence | Judgment | Supporting  evidence | Judgment | Supporting  evidence | Judgment | Supporting  evidence | Judgment | Supporting  evidence | Judgment | Supporting  evidence |
| Sherman[1]/  2018 | Low | Computer-generated  Randomization list | Low | ‘Participants were randomly assigned using the Qualtrics (Seattle, WA) randomizer function’ | Low | Participants and researchers were  blinded to the group allocation.  outcomes  questionnaires filled on online system | Low | Double blind method was used | Low | Attrition and reasons for attrition  reported and similar across  groups. | Low | Study protocol and trial  registration available and all  pre-specified outcomes  reported in pre-specified way | Low | No other bias was apparent |
| Bandani Susan[2]/  2022 | NC | Insufficient  information  to permit  judgement | NC | Insufficient  information  to permit  judgement | Low | Participants, assistant researcher, and statistical analyst were blinded to group allocation | NC | Insufficient information to permit  judgement | Low | Attrition and reasons for attrition  reported and similar across  groups | Low | Study protocol and trial  registration available and all  pre-specified outcomes  reported in pre-specified way | Low | No other bias was apparent |
| Graboyes[3]/  2023 | Low | Block Randomization Design | Low | ‘using a permuted block randomization design with block sizes of 4 or 6’ | Low | Participants and researchers were  blinded to the group allocation | Low | Outcome assessor blinding was not clear but did not affect the results | Low | No patients were lost to follow-up | Low | Study protocol and trial  registration available and all  pre-specified outcomes  reported in pre-specified way | Low | No other bias was apparent |

Abbreviations:NC:Not clear

**References**

1. Sherman KA, Przezdziecki A, Alcorso J, Kilby CJ, Elder E, Boyages J, et al. Reducing body image-related distress in women with breast cancer using a structured online writing exercise: results from the my changed body randomized controlled trial. J Clin Oncol. 2018;36(19):1930-40. [[FREE Full text](https://www.sci-hub.ru/10.1200/JCO.2017.76.3318)][doi:[10.1200/JCO.2017.76.3318](https://ascopubs.org/doi/10.1200/JCO.2017.76.3318?url_ver=Z39.88-2003&rfr_id=ori:rid:crossref.org&rfr_dat=cr_pub%20%200pubmed)][Medline:[29688834](https://pubmed.ncbi.nlm.nih.gov/29688834/)]
2. Bandani-Susan B, Montazeri A, Haghighizadeh MH, Araban M. The effect of mobile health educational intervention on body image and fatigue in breast cancer survivors: a randomized controlled trial. Ir J Med Sci 2022 Aug;191(4):1599-1605. [doi: [10.1007/s11845-021-02738-5](https://link.springer.com/article/10.1007/s11845-021-02738-5)] [Medline:[34370166](https://pubmed.ncbi.nlm.nih.gov/34370166/)]
3. Graboyes EM, Kistner-Griffin E, Hill EG, Maurer S, Balliet W, Williams AM, et al. Mechanism underlying a brief cognitive behavioral treatment for head and neck cancer survivors with body image distress. Support Care Cancer. 2023;32(1):32.[doi: [10.1007/s00520-023-08248-7](https://link.springer.com/article/10.1007/s00520-023-08248-7)][Medline:[38102496](https://pubmed.ncbi.nlm.nih.gov/38102496/)]
