# Supplementary material for: Effect of eHealth Interventions on Body Image of Patients With Cancer: Systematic Review
Source: J Med Internet Res. 2025 Jan 9;27:e55564. doi: 10.2196/55564 (PMC11757978; doi:10.2196/55564)
Supplement: Multimedia Appendix 4 [file jmir_v27i1e55564_app4.docx]

| Author/  Year | ① | | ② | | ③ | | ④ | | ⑤ | | ⑥ | | ⑦ | | ⑧ | | ⑨ | |
| --- | --- | --- | --- | --- | --- | --- | --- | --- | --- | --- | --- | --- | --- | --- | --- | --- | --- | --- |
|  | Judgment | Supporting  evidence | Judgment | Supporting  evidence | Judgment | Supporting  evidence | Judgment | Supporting  evidence | Judgment | Supporting  evidence | Judgment | Supporting  evidence | Judgment | Supporting  evidence | Judgment | Supporting  evidence | Judgment | Supporting  evidence |
| Weiner  [1]/  2023 | Y | The causal relationship in the study was clearly articulated | NC | Insufficient information to  permit judgement | NA | Single arm feasibility trial | Y | Self before and after control trial | Y | Physical activity and quality of life were measured at multiple time points using multiple scales | Y | Intentionality treatment analysis and last observation carried forward were used | Y | The same scale was used to evaluate the outcome indicators before and after the intervention | Y | Physical activity and quality of life measures have good feasibility and acceptability | Y | The data analysis method was appropriate |
| Chang  [2]/  2022 | Y | The causal relationship in the study was clearly articulated | N | There was statistical difference in age between the groups, and no statistical methods were used to control it | Y | Except for the intervention to be validated, the other measures received by each group were the same | Y | Non-randomized quasi-experimental study | N | Multiple time points were measured, but mental health, body image, and self-efficacy were measured with a single scale | N | Missing datas were reported, but no action was taken to address | Y | The same scale was used to evaluate the outcome indicators of each group | Y | Mental health, self-efficacy, and body image measures have good reliability | Y | The data analysis method was appropriate |
| Brkic[3]/  2024 | Y | The causal relationship in the study was clearly articulated | Y | Baseline comparison between groups | N | There were differences in other measures accepted by the two groups | Y | Non-randomized quasi-experimental study | Y | body image and distress were measured at multiple time points using multiple scales | Y | Appropriate statistical analyses were performed for the released population | Y | The same scale was used to evaluate the outcome indicators before and after the intervention | Y | The scales used were mature and reliable | Y | The data analysis method was appropriate |
| Grossert[4]/  2023 | Y | The causal relationship in the study was clearly articulated | NC | Insufficient information to  permit judgement | NA | pre-post convergent parallel design | Y | Self before and after control trial  Non-randomized quasi-experimental study | Y | Psychological health and body image were measured at multiple time points using multiple scales | Y | Appropriate statistical analyses were performed for the released population | Y | The same scale was used to evaluate the outcome indicators before and after the intervention | Y | The scale used in this study has good reliability and validity | Y | The data analysis method was appropriate |

Abbreviations:Y:Yes;N:No;NA: Not applicable;NC:Not clear

**References**

1. Weiner LS, Nagel S, Irene Su H, Hurst S, Levy SS, Arredondo EM, et al. A remotely delivered, peer-led intervention to improve physical activity and quality of life in younger breast cancer survivors. J Behav Med 2023 Aug;46(4):578-593. [[FREE Full text](https://www.ncbi.nlm.nih.gov/pmc/articles/PMC9735111/)][doi: [10.1007/s10865-022-00381-8](https://pubmed.ncbi.nlm.nih.gov/36479658/)][Medline:[36479658](https://pubmed.ncbi.nlm.nih.gov/36479658/)]
2. Chang YC, Chiu CF, Wang CK, Wu CT, Liu LC, Wu YC. Short-term effect of internet-delivered mindfulness-based stress reduction on mental health, self-efficacy, and body image among women with breast cancer during the COVID-19 pandemic. Front Psychol 2022 Oct ;13:949446. [[FREE Full text](https://www.ncbi.nlm.nih.gov/pmc/articles/PMC9640939/)][doi: [10.3389/fpsyg.2022.949446](https://www.frontiersin.org/journals/psychology/articles/10.3389/fpsyg.2022.949446/full)][Medline:[36389600](https://pubmed.ncbi.nlm.nih.gov/36389600/)]
3. Brkic E, Prichard I, Daly A, Dudley S, Beatty L. Testing the efficacy of a brief online writing intervention on body image and distress in female cancer survivors. Patient Educ Couns. 2024;127:108356. [doi: [10.1016/j.pec.2024.108356](https://www.sciencedirect.com/science/article/pii/S0738399124002234?via%3Dihub)][Medline:[38944983](https://pubmed.ncbi.nlm.nih.gov/38944983/)]
4. Grossert A, Meffert C, Hess V, Rochlitz C, Pless M, Hunziker S, et al. Group-based body psychotherapy improves appreciation of body awareness in post-treatment cancer patients: a non-randomized clinical trial. Front Psychol. 2023;14:956493. [[Free Full text](https://www.ncbi.nlm.nih.gov/pmc/articles/PMC10117640/)][doi :[10.3389/fpsyg.2023.956493](https://www.frontiersin.org/journals/psychology/articles/10.3389/fpsyg.2023.956493/full)][Medline:[36479658](https://pubmed.ncbi.nlm.nih.gov/37089722/)]
