# Supplementary material for: Effect of eHealth Interventions on Body Image of Patients With Cancer: Systematic Review
Source: J Med Internet Res. 2025 Jan 9;27:e55564. doi: 10.2196/55564 (PMC11757978; doi:10.2196/55564)
Supplement: Multimedia Appendix 5 [file jmir_v27i1e55564_app5.docx]

| Section/topic | # | Checklist item | Reported on paragraph  # |
| --- | --- | --- | --- |
| TITLE | | | |
| Title | 1 | Identify the report as a systematic review | 1 |
| ABSTRACT | | | |
| Structured summary | 2 | Provide a structured summary including, as applicable: objectives; data sources; study eligibility criteria, participants, and interventions; study appraisal and synthesis methods; results; limitations; conclusions and implications of key findings; systematic review registration number. | 2-7 |
| INTRODUCTION | | |  |
| Rationale | 3 | Describe the rationale for the review in the context of what is already known. | 8-12 |
| Objectives | 4 | Provide an explicit statement of the objective(s)or question(s) the review addresses. | 12 |
| METHODS | | | |
| Protocol and  registration | 5 | Indicate if a review protocol exists, if and where it can be accessed (e.g., Web address), and, if available, provide registration information including registration number. | 13 |
| Eligibility criteria | 6 | Specify the inclusion and exclusion criteria for the review and how studies were grouped for the syntheses | 15-16 |
| Information sources | 7 | Specify all databases, registers, websites, organisations, reference lists and other sources searched or consulted to identify studies. Specify the date when each source was last searched or consulted. | 14 |
| Search | 8 | Present the full search strategies for all databases, registers and websites, including any filters and limits used. | 14 |
| Study selection | 9 | Specify the methods used to decide whether a study met the inclusion criteria of the review, including how many reviewers screened each record and each report retrieved, whether they worked independently, and if applicable, details of automation tools used in the process. | 17 |
| Data collection  process | 10 | Specify the methods used to collect data from reports, including how many reviewers collected data from each report, whether they worked independently, any processes for obtaining or confirming data from study investigators, and if applicable, details of automation tools used in the process. | 18 |
| Data items | 11 | List and define all other variables for which data were sought (e.g. participant and intervention characteristics, funding sources).Describe any assumptions made about any missing or unclear information. | 18 |
| Risk of bias in  individual studies | 12 | Specify the methods used to assess risk of bias in the included studies, including details of the tool(s) used, how many reviewers assessed each study and whether they worked independently, and if applicable, details of automation tools used in the process. | 19 |
| Summary measures | 13 | Specify for each outcome the effect measure(s) (e.g. risk ratio, mean difference) used in the synthesis or presentation of results. | 20 |
| Synthesis of results | 14 | Describe the processes used to decide which studies were eligible for each synthesis (e.g. tabulating the study intervention characteristics and comparing against the planned groups for each synthesis .Describe any methods used to synthesis results and provide a rationale for the choice(s) | 20 |
| RESULTS | | | |
| Study selection | 17 | Describe the results of the search and selection process, from the number of records identified in the search to the number of studies included in the review, ideally using a flow diagram. | 21 |
| Study characteristics | 18 | Cite each included study and present its characteristics | 21-24 |
| Risk of bias within studies | 19 | Present assessments of risk of bias for each included study | 32 |
| Results of individual studies | 20 | For all outcomes, present, for each study summary statistics for each group (where appropriate) using structured tables or plots. | 21 |
| Synthesis of results | 21 | Present results of all statistical syntheses conducted | 25-31 |
| Risk of bias across studies | 22 | Present assessments of risk of bias due to missing results (arising from reporting biases) for each synthesis assessed. | 32 |
| DISCUSSION | | | |
| Summary of evidence | 24 | Provide a general interpretation of the results in the context of other evidence. | 33-36 |
| Limitations | 25 | Discuss any limitations of the evidence included in the review.Discuss any limitations of the evidence included in the review. | 37 |
| Conclusions | 26 | Discuss implications of the results for practice, policy, and future research | 38 |
| FUNDING | | | |
| Funding | 27 | Describe sources of financial or non-financial support for the review, and the role of the funders or sponsors in the review. | 39 |
| Competing interests | 28 | Declare any competing interests of review authors. | 40 |
